# The Relationship Between Pre-existing Coronary Heart Disease and Cognitive Impairment Is Partly Explained by Reduced Left Ventricular Ejection Fraction in the Subjects Without Clinical Heart Failure: A Cross-Sectional Study

**DOI:** 10.3389/fnhum.2022.835900

**Published:** 2022-05-11

**Authors:** Suhang Shang, Ziyu Liu, Jinying Gao, Jin Wang, Wenhui Lu, Yulang Fei, Binyan Zhang, Baibing Mi, Pei Li, Louyan Ma, Yu Jiang, Chen Chen, Liangjun Dang, Jie Liu, Qiumin Qu

**Affiliations:** ^1^Department of Neurology, The First Affiliated Hospital of Xi’an Jiaotong University, Xi’an, China; ^2^Department of Neurology, The People’s Hospital of Ankang, Ankang, China; ^3^Medical College, Xijing University, Xi’an, China; ^4^Department of Epidemiology and Health Statistics, School of Public Health, Xi’an Jiaotong University Health Science Center, Xi’an, China; ^5^The Assisted Reproductive Technology Center, Northwest Women’s and Children’s Hospital, Xi’an, China; ^6^The Second Department of Geriatrics, Xi’an No 9 Hospital, Xi’an, China; ^7^Department of Neurology, The Second Affiliated Hospital of Xi’an Jiaotong University, Xi’an, China

**Keywords:** coronary heart disease, cognitive impairment, left ventricular ejection fraction, mini-mental state examination, cross-sectional study

## Abstract

**Background:**

Coronary heart disease (CHD) is closely associated with cognitive impairment, especially in severe cases of heart failure. However, it is unclear whether cardiac systolic function plays a role in the relationship between pre-existing CHD and cognitive impairment in subjects without clinical heart failure.

**Methods:**

In total, 208 subjects from the First Affiliated Hospital of Xi’an Jiaotong University were recruited from June 2014 to January 2015, and were divided into CHD (*n* = 118) and non-CHD (*n* = 90) groups according to the inclusion and exclusion criteria. The global cognitive function of all subjects was assessed by the Mini-Mental State Examination (MMSE) and cognitive impairment was defined as the score lower than the cutoff value. Left ventricular ejection fraction (LVEF) was measured using transthoracic echocardiograms. The relationship among pre-existing CHD, LVEF, and cognitive impairment was analyzed by multivariate logistic regression.

**Results:**

In total, 34 subjects met the criteria of cognitive impairment. Univariate analysis showed that the cognitive impairment prevalence in the CHD group was significantly higher than that in the non-CHD group (22.0 vs. 8.9%, *p* = 0.011). Multivariate logistic analysis revealed that CHD was significantly associated with a higher risk of cognitive impairment (odds ratio [*OR*] = 3.284 [95% *CI*, 1.032–10.450], *p* = 0.044) after adjusting for confounds except for LVEF. However, the OR of CHD decreased (*OR* = 2.127 [95% *CI*, 0.624–7.254], *p* = 0.228) when LVEF was further corrected as a continuous variable, and LVEF was negatively associated with the risk of cognitive impairment (*OR* = 0.928 [95% *CI*, 0.882–0.976], *p* = 0.004).

**Conclusion:**

Pre-existing CHD is associated with a higher risk of cognitive impairment, and such an association can be considerably explained by reduced LVEF. An impaired cardiac systolic function may play a key role in the relationship between CHD and cognitive impairment among patients with pre-heart failure conditions.

## Introduction

Coronary heart disease (CHD) and cognitive impairment are common diseases in older adults (Zlokovic et al., 2020). The association between the CHD and cognitive impairment is close (Roberts et al., 2013; Schievink et al., 2017; Liang et al., 2021), and is partly explained by shared vascular risk factors (Zlokovic et al., 2020). However, some research shows that the association still exists after correcting for common risk factors (Liang et al., 2021), which suggests that in addition to common risk factors, there are other factors mediating the association between them.

Previous studies have testified that cardiac systolic function parameters were associated with cerebral blood flow (Roy et al., 2017), brain volume (Sabayan et al., 2015), and cognitive function (Jefferson et al., 2015). Therefore, cardiac improvement therapy in patients with heart failure can improve cognitive performance (Duncker et al., 2015). As described above, the cardiac function, which ranges from normal cardiac function to the higher stage of heart failure in the subjects with CHD, could be the most significant confounding factor. However, the subjects with clinical heart failure were not excluded in most previous studies (Roberts et al., 2013; Jefferson et al., 2015; Liang et al., 2021), and it is still uncertain whether CHD increases the risk for cognitive impairment among patients with pre-heart failure conditions.

This study aimed to research the relationship between pre-existing CHD and cognitive impairment in the subjects without clinical heart failure, and whether the association of them can be explained by reduced left ventricular ejection fraction (LVEF), the latter is widely applied to reflect the cardiac systolic function in clinical.

## Materials and Methods

### Study Design and Participants

This is a cross-sectional study to research the relationship among CHD, LVEF, and cognitive impairment. Subjects from the Department of Cardiology or Neurology, the First Affiliated Hospital of Xi’an Jiaotong University were recruited from June 2014 to January 2015. Inclusion criteria were as follows: (1) ≥50 years old; (2) for the CHD group: confirmed CHD history for more than 6 months, but no acute coronary event in last 6 months; for the non-CHD group: admitted to hospital because of increased blood pressure (BP), vertigo, or dizzy; (3) accepted the transthoracic echocardiograms; and (4) agreed to participate in the study and were able to finish the survey. The exclusion criteria were as follows: (1) subjects who suffered from other severe heart diseases, such as valvular heart disease, dilated cardiomyopathy, and severe arrhythmia (persistent atrial flutter, persistent atrial fibrillation, second and third-degree atrioventricular block, sustained ventricular tachycardia, and sick sinus syndrome); (2) newly diagnosed acute coronary syndrome during hospitalization; (3) clinical heart failure, or the LVEF < 40%; (4) individuals who suffered from diabetes, stroke, Parkinson’s disease, intracranial space-occupying lesion, traumatic brain injury, epilepsies, uncorrected hypothyroidism, and vitamin B_12_ deficiency; (5) carotid artery ultrasound examination revealed severe carotid artery stenosis (stenosis rate > 50%); and (6) other serious systematic diseases.

All subjects accepted the general examination, such as electrocardiogram (ECG), cardiac enzymes, high sensitive cardiac Troponin I, ultrasonic cardiogram, and carotid artery ultrasonography; subjects with vertigo or dizziness accepted the imaging examination of the head (CT or MRI); and subjects with suspected acute coronary syndrome accepted coronary angiography.

**FIGURE 1:**
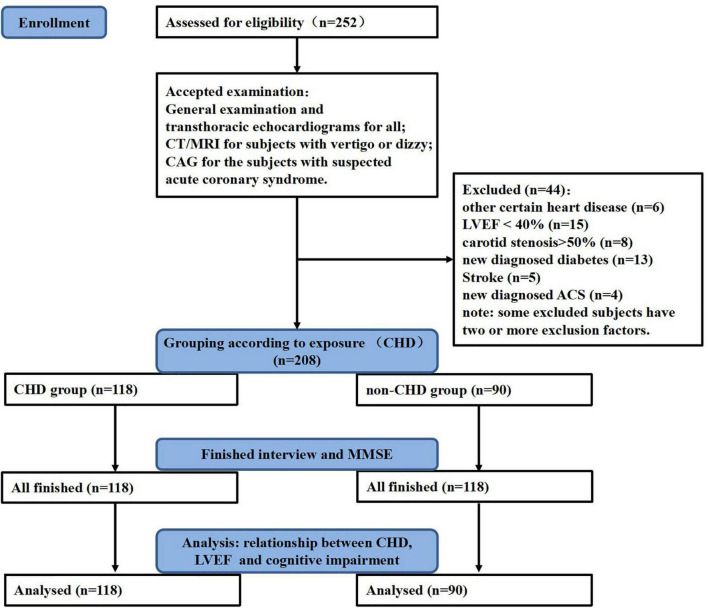
Study flowchart. General examination includes an electrocardiogram (ECG), cardiac enzymes, high sensitive cardiac Troponin I, carotid artery ultrasonography, and other routine examination. CHD, coronary heart disease; MMSE, mini-mental state examination; LVEF, left ventricular ejection fraction; CAG, coronary angiography; CT, computerized tomography; MRI, magnetic resonance imaging; ACS, acute coronary syndrome.

To start with, 252 subjects were enrolled in this study after being assessed for eligibility. Thereafter, those with one or more exclusion criteria were excluded. In total, 44 subjects were excluded and 208 subjects were left. Detail information was shown in Figure 1. Finally, 118 subjects were included in the CHD group and 90 subjects in the non-CHD group. General information, such as demographic information, lifestyles, comorbidities, physical examination parameters, biochemical test parameters, and drug use before admission, was collected through face-to-face interviews, and cognitive function was tested by Mini-Mental State Examination (MMSE). Written informed consent was obtained from all participants, and the study was approved by the Medical Ethics Committee of the First Affiliated Hospital of Xi’an Jiaotong University.

### Diagnosis of Coronary Heart Disease

All subjects passed a general cardiac examination after enrollment. For those with suspected CHD, CAG was also conducted. Cardiac physicians made the final diagnosis of CHD on the basis of the guidelines for cardiac disease (Hunt et al., 2005; Anderson et al., 2007; Antman et al., 2008; King et al., 2008; Fihn et al., 2014). In this study, CHD history means stable angina, acute coronary syndrome (unstable angina or myocardial infarction), coronary artery stenosis on angiography, or prior percutaneous coronary intervention not before 6 months ago as documented by medical records or a key medical report.

### Cardiac Systolic Function

Transthoracic echocardiograms were performed using the GE Vivid 7.0 ultrasound diagnostic instrument by trained echocardiographers from the Department of the Echocardiography, the First Affiliated Hospital of Xi’an Jiaotong University. LVEF was calculated with the Teichholtz formula to reflect the cardiac systolic function (Sun et al., 2001). The parameters were measured three times and then averaged.

### Cognitive Function Evaluation

Global cognitive function was assessed using the MMSE in a separate quiet room (Folstein et al., 1975). Cognitive impairment was defined as the MMSE score lower than the cutoff value specified for education level: ≤17 for the uneducated, ≤20 for the educated at the level of primary school, and ≤24 for those educated at or above the level of junior high school (Katzman et al., 1988).

### Covariates

The demographic information, lifestyles, comorbidities, physical examination parameters, biochemical test parameters, and drug use before admission were collected. Most of the information collected is shown in Table 1. The information of educational levels was collected as a continuous variable (years of education), and then categorized as illiterate (uneducated), primary school (≤6 years of education), and junior high school or above (>6 years of education) (Katzman et al., 1988). Physical exercises were divided into regular exercises (exercises ≥ 3 times/week and ≥ 30 min/time, or heavy manual workers) or lack of exercise. Hypertension was defined as either high BP (systolic BP ≥ 140 mmHg or diastolic BP ≥ 90 mmHg) or sure history of hypertension (James et al., 2014). Dyslipidemia was based on the self-report, and biochemical tests of the blood lipid were also completed. Besides, fasting blood glucose and cervical vessels ultrasound examination were conducted for every subject. All the biochemical tests were operated by the biochemical laboratory of the First Affiliated Hospital of Xi’an Jiaotong University. The drug use (antihypertensive drugs, antidiabetic drugs, lipid-lowering agents, and antiplatelet drugs) before admission was self-reported.

**TABLE 1:** Demographic data and clinical characteristics of the study population.

Variables	Total	CHD	Non-CHD	*P*
	(*n* = 208)	(*n* = 118)	(*n* = 90)	
Male, *n* (%)	117 (56.3)	74 (62.7)	43 (47.8)	0.031
Age, Mean (SD), year	65.68 (8.76)	66.03 (8.44)	65.22 (9.19)	0.514
Formal education, *n* (%)				0.834
Uneducated	21 (10.1)	12 (10.2)	9 (10.0)	
Primary school	18 (8.7)	9 (7.6)	9 (10.0)	
High school or above	169 (81.3)	97 (82.2)	72 (80.0)	
Years of education, Mean (SD), year	10.13 (4.57)	10.11 (4.41)	10.14 (4.80)	0.957
LPA, *n* (%)	37 (17.8)	28 (23.7)	9 (10.0)	0.010
Tobacco use, *n* (%)	83 (39.9)	52 (44.1)	31 (34.4)	0.160
Alcohol consumption, *n* (%)	52 (25.0)	32 (27.1)	20 (22.2)	0.419
Hypertension, *n* (%)	139 (66.8)	82 (69.5)	57 (63.3)	0.350
Dyslipidemia, *n* (%)	82 (39.4)	52 (44.1)	30 (33.3)	0.117
Antihypertensive drugs, *n* (%)	110 (52.9)	66 (55.9)	44 (48.9)	0.313
Antiplatelet drugs, *n* (%)	44 (21.2)	41 (34.7)	3 (3.3)	<0.001
Statins, *n* (%)	33 (15.9)	31 (26.3)	2 (2.2)	<0.001
SBP, Mean (SD), mmHg	134.60 (20.09)	133.64 (21.18)	135.86 (18.62)	0.434
DBP, Mean (SD), mmHg	76.29 (12.65)	74.81 (12.50)	78.24 (12.64)	0.052
BMI, Mean (SD), kg/m^2^	24.32 (3.33)	24.56 (3.42)	24.00 (3.20)	0.235
FBG, Median (P25,	5.17	5.17	5.17	0.865
P75), mmol/L	(4.59, 6.43)	(4.62, 6.58)	(4.52, 6.24)	
TG, Median (P25,	1.32	1.25	1.40	0.087
P75), mmol/L	(0.95, 1.75)	(0.95, 1.60)	(0.97, 1.86)	
TC, Mean (SD), mmol/L	3.93 (0.95)	3.79 (0.89)	4.12 (0.99)	0.014
LDL, Mean (SD), mmol/L	2.27 (0.81)	2.19 (0.75)	2.37 (0.86)	0.106
HDL, Mean (SD), mmol/L	1.06 (0.26)	1.01 (0.23)	1.13 (0.29)	0.001
HCY, Median (P25,	16.0	16.9	14.9	0.009
P75), mmol/L	(13.1, 21.2)	(13.9, 25.2)	(12.3, 19.8)	
LVEF, Median (P25, P75), %	66(62,71)	66(60,70)	67(65,72)	<0.001
MMSE score, Median (P25, P75)	27 (25, 29)	27 (24, 28)	27 (25, 29)	0.224
CogI, *n* (%)	34 (16.3)	26 (22.0)	8 (8.9)	0.011

*CHD, coronary heart disease; Non-CHD, non-coronary heart disease; LPA, lack of physical activity; BMI, body mass index; FBG, fasting blood glucose; TG, triglycerides; TC, total cholesterol; LDL, low-density lipoprotein; HDL, high-density lipoprotein; HCY, homocysteine; LVEF, left ventricular ejection fraction; MMSE, mini-mental state examination; CogI, cognitive impairment.*

### Statistical Analysis

Statistical analysis was performed using SPSS 25.0 statistical software. A *p* less than 0.05 was considered significant, and all tests were two sided. The characteristics of the subjects were reported as mean ± SD for normally distributed continuous variables, as the median (25 percentile and 75 percentile) for skewed continuous variables, and as numbers (percentages) for categorical variables. In univariate analysis, *t*-tests, rank tests, χ^2^ tests were performed according to the data types and distribution. In multivariate analysis, logistic regression models were performed to correct for confounding factors. In the logistic regression models, cognitive impairment (yes or no) was the dependent variable, and the CHD (yes or no) and confounding factors were independent variables.

In multivariate analysis, we analyzed the relationship between cognitive impairment and CHD after correcting for most confounds collected in this study except LVEF (Model 1 and Model 2). In detail, age, gender, and years of education were corrected in Model 1. Lack of physical activities, tobacco use, alcohol consumption, hypertension, body mass index, triglycerides, total cholesterol, low-density lipoprotein, high-density lipoprotein, and homocysteine were further corrected in Model 2. Then, the LVEF was further corrected in Model 3 to analyze their correlation.

## Results

### Demographics and Clinical Characteristics

In total, 208 subjects were included in this study, 118 subjects in the CHD group and 90 subjects in the non-CHD group. The mean age of the sample was 65.68 ± 8.76 years (range 50–86 years), and 117 (56.3%) of them were men. The MMSE score had a skewed distribution [27 (25, 29)], and 34 subjects met the criterion of cognitive impairment. The LVEF value also showed a skewed distribution [66 (62, 71)]. The detailed clinical information about the population was presented in Table 1.

**FIGURE 2:**
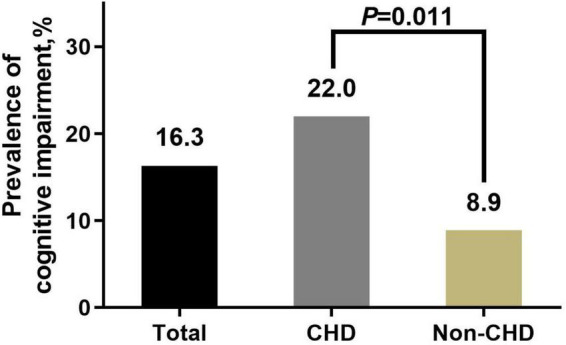
Prevalence of cognitive impairment according to CHD. CHD, coronary heart disease.

The prevalence of cognitive impairment was 16.3% in this sample. Univariate analysis showed that the cognitive impairment prevalence in the CHD group was significantly higher than that in the non-CHD group (22.0 vs. 8.9%, *p* = 0.011) (Figure 2). Detailed information of differences in covariates between the cognitive impairment group and the normal cognition group is shown in Table 2.

**TABLE 2:** Differences in covariates between the cognitive impairment group and the normal cognition group in the total population.

	CogI (*n* = 34)	NC (*n* = 174)	*P*
Male, *n* (%)	17 (50.0)	100 (57.5)	0.422
Age, Mean (SD), year	70.50 (8.23)	64.74 (8.57)	<0.001
Formal education, *n* (%)			<0.001
Uneducated	11 (32.4)	10 (5.7)	
Primary school	2 (5.9)	16 (9.2)	
High school or above	21 (61.8)	148 (85.1)	
Years of education, Mean (SD), year	7.35 (5.76)	10.67 (4.10)	0.003
LPA, *n* (%)	10 (29.4)	27 (15.5)	0.053
Tobacco use, *n* (%)	15 (44.1)	68 (39.1)	0.583
Alcohol consumption, *n* (%)	6 (17.6)	46 (26.4)	0.279
Hypertension, *n* (%)	31 (91.2)	108 (62.1)	0.001
Dyslipidemia, *n* (%)	14 (41.2)	68 (39.1)	0.819
CHD, *n* (%)	26 (76.5)	92 (52.9)	0.011
Antihypertensive drugs, *n* (%)	18 (52.9)	92 (52.9)	0.994
Antiplatelet drugs, *n* (%)	10 (29.4)	34 (19.5)	0.197
Statins, *n* (%)	6 (17.6)	27 (15.5)	0.756
SBP, Mean (SD), mmHg	125.45 (17.12)	136.39 (20.19)	0.003
DBP, Mean (SD), mmHg	70.87 (10.12)	77.35 (12.84)	0.006
BMI, Mean (SD), kg/m^2^	23.38 (3.13)	24.50 (3.35)	0.072
FBG, Median (P25, P75), mmol/L	5.58(4.85,6.83)	5.17(4.56,6.24)	0.107
TG, Median (P25, P75), mmol/L	1.32(1.03,1.59)	1.33(0.95,1.77)	0.955
TC, Mean (SD), mmol/L	3.90 (0.87)	3.94 (0.96)	0.831
LDL, Mean (SD), mmol/L	2.36 (0.76)	2.25 (0.82)	0.486
HDL, Mean (SD), mmol/L	0.98 (0.20)	1.07 (0.27)	0.069
HCY, Median (P25,	18.15	15.70	0.106
P75), mmol/L	(13.88, 27.93)	(13.00, 21.00)	
LVEF, Median (P25, P75), %	61(49,65)	67(64,72)	<0.001

*CogI, cognitive impairment; NC, normal cognition; LPA, lack of physical activity; CHD, coronary heart disease; SBP, systolic blood pressure; DBP, diastolic blood pressure; BMI, body mass index; FBG, fasting blood glucose; TG, triglycerides; TC, total cholesterol; LDL, low-density lipoprotein; HDL, high-density lipoprotein; HCY, homocysteine; LVEF, left ventricular ejection fraction.*

### Relationship Between Coronary Heart Disease and Cognitive Impairment After Correcting for Other Confounds but Left Ventricular Ejection Fraction

Logistic regression models were established to correct the confounding factors. In the models, cognitive impairment (yes or no) was set as dependent variable, and the factors adjusted in different models are shown in Table 3. CHD was prominently associated with a higher risk of cognitive impairment (Model 1: odds ratio [*OR*] = 3.425 [95% *CI*, 1.357–8.644], *p* = 0.009 and Model 2: *OR* = 3.284 [95% *CI*, 1.032–10.450], *p* = 0.044). The detailed information of the Model 1 and Model 2 have been described in the Statistical Analysis Section.

**TABLE 3:** Relationship between coronary heart disease (CHD) and cognitive impairment after correcting for confounds.

Variables	B	S.E.	Wald	OR	95% CI	*P*
**Model 1**						
CHD	1.231	0.472	6.792	3.425	1.357-8.644	0.009
**Model 2**						
CHD	1.189	0.591	4.055	3.284	1.032-10.450	0.044
**Model 3**						
CHD	0.755	0.626	1.454	2.127	0.624-7.254	0.228
LVEF	−0.075	0.026	0.504	0.928	0.882-0.976	0.004

*Model 1: adjusted for age, gender, and years of education; Model 2: adjusted for age, gender, years of education, lack of physical activity, tobacco use, alcohol consumption, hypertension, body mass index, triglycerides, total cholesterol, low-density lipoprotein, high-density lipoprotein, and homocysteine; Model 3: adjusted for variables in Model 2 plus LVEF; LVEF were considered continuous variables in the Model 3.*

### Relationship Between Coronary Heart Disease and Cognitive Impairment After Correcting for Left Ventricular Ejection Fraction

To research the impact of LVEF on the relationship between CHD and cognitive impairment, we established Model 3 to further correct for LVEF value based on Model 2. Compared with Model 2, *OR* of CHD in Model 3 decreased and the relationship between CHD and cognitive impairment was not so prominent as in Model 2 (Model 3: *OR* = 2.127 [95% *CI*, 0.624–7.254], *p* = 0.228). Besides, LVEF was negatively associated with cognitive impairment (*OR* = 0.928 [95% *CI*, 0.882–0.976], *p* = 0.004), which indicated that the positive relationship between CHD and cognitive impairment could be considerably explained by the reduction of LVEF (Table 3).

## Discussion

This study showed that pre-existing CHD significantly increased the risk of cognitive impairment in subjects without clinical heart failure, and the strength of this association decreased greatly when the LVEF was further corrected. It indicates that reduced LVEF plays a key role in the relationship between CHD and cognitive impairment among patients with pre-heart failure conditions. However, the exact cut-off value of LVEF that causes an increased risk of cognitive impairment is difficult to define in this study because of the small sample size.

Whether the effect of LVEF is a mediate effect or a moderate effect is a question that needs to be discussed. A mediate effect is when the association between exposure and outcome is mediated through mediator variables. A moderate effect is when a moderator variable can change the strength of the association between exposure and outcome. The results of Model 2 and Model 3 (as shown in Table 3) have supported the effect of LVEF on the relationship between CHD and cognitive impairment, which is a mediate effect. Besides, a CHD × LVEF term was also included in the models in the analysis (results not shown), and no significant moderate effect was found. In summary, the effect of LEVF is considered to be a mediate effect.

Our results agree with many previous studies which have testified the association between CHD history and increased risk of cognitive impairment. Beeri et al. (2006) examined the correlation between postmortem AD neuropathology and autopsy-verified cardiovascular disease, and showed that the degree of coronary artery disease was independently associated with the cardinal neuropathological lesions of AD. Schievink et al. (2017) found that during 12 years of follow-up, both prevalent and incident CHDs were associated with more decline in multiple cognitive domains. However, these studies neither excluded the subject with clinical heart failure nor examined the impact of cardiac function on this relation.

Previous studies focusing on the relationship between cardiac function and cognitive impairment were mainly conducted in general population. A prospective cohort study with an average of 7.7 years of follow-up showed that a lower cardiac index assessed by cardiac magnetic resonance imaging (MRI) was linked with an increased risk for the development of dementia and AD in the general population, and the result did not change even when all of the subjects with clinically prevalent cardiovascular disease were excluded (Jefferson et al., 2015). Another study indicated that the N-terminal pro-brain natriuretic peptide, a serum marker of cardiac function, is a predictor of cognitive decline in the oldest population (van Vliet et al., 2014). In addition, high baseline concentrations of high-sensitivity cardiac troponin T (a potential marker for subclinical myocardial damage that may cause inadequate left ventricular function) were found associated with lower cognitive test scores at baseline and increased dementia hospitalization risk during follow-up in community-based populations without coronary heart disease, myocardial infarction, heart failure, or stroke at baseline (Schneider et al., 2014). However, this study discovered that pre-existing CHD subjects without clinical heart failure were associated with poorer cognitive function, and reduction of LVEF could considerably explain this association.

Epidemiology data suggested that cardiac dysfunction (mainly reflected by reduced cardiac output) was associated with smaller brain volumes (Kumar et al., 2015) and white matter lesions (Roy et al., 2017), both of which could be possible ways leading to cognitive impairment. Some studies have also found that these cerebral changes may be caused by a reduction of cerebral blood flow which may be related to the systemic blood flow (Jefferson et al., 2011). Present studies have found that chronic cerebral hypoperfusion could result in a series of cerebral changes, such as neuronal injury and death, blood-brain barrier disruption, cerebrovascular pathologies, and progression of AD pathology (Seo et al., 2013; Park et al., 2019; Wang et al., 2019). All of the above may independently and collectively contribute to cognitive impairment. Hence, chronic cerebral hypoperfusion may be a key point connecting cardiac dysfunction and cognitive impairment (Xu et al., 2021). Besides, common vascular risk factors, such as hypertension can also impair the cerebral vascular autoregulation function (Iadecola et al., 2016), which can make the subjects more susceptible to the systemic blood flow disturbance. In summary, we make the hypothesis that pre-existing CHD leads to a reduction of cardiac systolic function, which contributes to systemic blood flow disturbance and may further impair cerebral hemodynamics, hence becoming a possible path influencing the cognitive function in a group with coronary artery disease (MacIntosh et al., 2015). Notably, it is very challenging to testify the relationship between cerebral hypoperfusion and cognitive results in clinical research due to the complexity of CBF regulation, with a few recent studies have found a roughly stable cerebral blood flow for mild-moderate heart failure patients (Erkelens et al., 2017). Therefore, some other possible mechanisms may coexist, for instance, similar pathological changes of a blood vessel in the heart, and brain tissue might also partly explain the link between CHD and cognitive impairment. Therefore, future studies in the animal models are necessary.

There are some strength in this study. First, participants are with relatively low diversity in the clinical manifestation and therapeutic management. The clinical characteristics of the CHD are diverse, with cardiac systolic function ranging from normal to advanced heart failure. The latter have been demonstrated as a risk factor of cognitive impairment. Besides, for those with acute myocardial infarction, the change of the cardiac systolic function may occur at once after myocardial infarction, but the effect of LVEF reduction on cognitive impairment may appear after a period of time because the change of cognitive function is a gradual process. The diversity in the subjects mentioned above may reduce the reliability of the result. Therefore, subjects with acute myocardial infarction, clinical heart failure or other severe heart diseases were all excluded in this study. Second, although subjects were recruited from hospital, the comparability between the CHD group and the non-CHD group was guaranteed by no significant difference of major confounding factors, especially the age and education. Third, our study found that the prominent relationship between pre-existing CHD and cognitive impairment can be considerably explained by a reduction in LVEF. Therefore, indicating that in clinical practice, cognition screening should also be considered in patients with CHD with reduced LVEF but not only with heart failure and early treatment to protect cardiac function may contribute to the maintenance of cognitive function. Last, for future relevant studies, LVEF is a necessary and important confounder, even in those without heart failure.

However, some limitations also existed. First, we failed to define an exact cut-off value of LVEF that causes an increased risk of cognitive impairment because of the small sample size. Moreover, our study utilized a cross-sectional design, and the LVEF values were tested after admission, but the LVEF values of the subjects in the CHD group when they were diagnosed with CHD were seriously missing, and most of the subjects in the non-CHD group have not accepted the echocardiography examination before, so we could not assess the change courses of LVEF and their impact. Third, the subjects with CHD accepted different treatments, for instance, just medication or combined with vascular interventions. Recruiting more subjects and conducting subgroup analysis according to different treatments are necessary for future research. Finally, the correction for multiple comparisons was not performed, considering that this was an exploratory study with a small sample size. Therefore, “significant” results based on this exploratory study should be further verified in future confirmatory studies.

## Conclusion

Pre-existing CHD is positively associated with cognitive impairment, and this association can be considerably explained by reduction of LVEF. The cardiac systolic function may play a key role in the relationship between CHD and cognitive impairment.

## Data Availability Statement

The datasets presented in this article are not readily available because the dataset contains patients’ other clinical information. Individuals interested in accessing the data must receive ethical approval from the Medical Ethics Committee of The First Affiliated Hospital of Xi’an Jiaotong University (xm3215@126.com). After achieving ethical approval, requests for a de-identified dataset from this study may be sent to QQ, quqiumin@xjtufh.edu.cn.

## Ethics Statement

The studies involving human participants were reviewed and approved by Medical Ethics Committee of The First Affiliated Hospital of Xi’an Jiaotong University. The patients/participants provided their written informed consent to participate in this study.

## Author Contributions

QQ and SS designed this study. SS did the statistical analysis and co-wrote the manuscript. ZL took part in the survey, assisted with the statistical analysis, and co-wrote the manuscript. JG, JW, WL, YF, BZ, BM, PL, LM, YJ, CC, LD, and JL took part in the survey and collected the samples. QQ provided quality control and technical guidance in all stages of the study. All authors read and approved the final manuscript.

## Conflict of Interest

The authors declare that the research was conducted in the absence of any commercial or financial relationships that could be construed as a potential conflict of interest.

## Publisher’s Note

All claims expressed in this article are solely those of the authors and do not necessarily represent those of their affiliated organizations, or those of the publisher, the editors and the reviewers. Any product that may be evaluated in this article, or claim that may be made by its manufacturer, is not guaranteed or endorsed by the publisher.
